# Synergistic Interactions of Methanolic Extract of *Acacia mearnsii* De Wild. with Antibiotics against Bacteria of Clinical Relevance

**DOI:** 10.3390/ijms13078915

**Published:** 2012-07-17

**Authors:** Olufunmiso O. Olajuyigbe, Anthony J. Afolayan

**Affiliations:** Phytomedicine Research Centre, Department of Botany, University of Fort Hare, Alice 5700, South Africa; E-Mail: funmijuyigbe12@yahoo.com

**Keywords:** *Acacia mearnsii*, checkerboard assay, drug-herbal interaction, multi-drug resistance, synergistic effects

## Abstract

With the emergence of multidrug-resistant organisms, combining medicinal plants with synthetic or orthodox medicines against resistant bacteria becomes necessary. In this study, interactions between methanolic extract of *Acacia mearnsii* and eight antibiotics were investigated by agar diffusion and checkerboard assays. The minimum inhibitory concentrations (MICs) of all the antibiotics ranged between 0.020 and 500 μg/mL while that of the crude extract varied between 0.156 and 1.25 mg/mL. The agar diffusion assay showed that extract-kanamycin combination had zones of inhibition ≥20 ± 1.0 mm in all the bacteria tested (100%), followed by extract-chloramphenicol (90%) > extract-ciprofloxacin = extract-tetracycline (70%) > extract-amoxicillin (60%) > extract-nalidixic acid (50%) > extract-erythromycin (40%) > extract-metronidazole (20%). The checkerboard showed synergistic interaction (61.25%), additivity/indifference (23.75%) and antagonistic (15%) effects. The synergistic interaction was most expressed by combining the extract with tetracycline, metronidazole, amoxicillin, ciprofloxacin, chloramphenicol and nalidixic acid against *E. coli* (ATCC 25922), erythromycin, metronidazole, amoxicillin, chloramphenicol and kanamycin against *S. aureus* (ATCC 6538), erythromycin, tetracycline, amoxicillin, nalidixic acid and chloramphenicol against *B. subtilis* KZN, erythromycin, metronidazole and amoxicillin against *E. faecalis* KZN, erythromycin, tetracycline, nalidixic acid and chloramphenicol against *K. pneumoniae* (ATCC 10031), erythromycin, tetracycline, metronidazole and chloramphenicol against *P. vulgaris* (ATCC 6830), erythromycin, tetracycline, amoxicillin and chloramphenicol against *S. sonnei* (ATCC 29930), metronidazole, amoxicillin and chloramphenicol against *E. faecalis* (ATCC 29212) and ciprofloxacin and chloramphenicol against *Proteus vulgaris* KZN. The synergistic interactions indicated that the bactericidal potentials of the antibacterial agents were improved and combining natural products with antibiotic could be potential sources for resistance-modifying agents useful against infectious multi-drug resistant bacteria.

## 1. Introduction

Infectious diseases are important causes of morbidity and mortality [1–3]. They remain the biggest killer of children and young adults as well as the second-leading cause of death worldwide [4,5] despite remarkable successes of antimicrobial drug generations of the third part of the last century. While host and pathogen interactions are complex and requires several effort to limit the spread of causal organisms [6], uncontained infections result in systemic inflammatory response syndrome that may progress into severe sepsis, septic shock, or mortality [7]. In recent years, bacterial infections caused by resistant strains have been increased dramatically [8]. Multiple drug resistance, an intrinsic and inevitable aspect of microbial survival that continually challenges human health [9], have resulted from the indiscriminate use of commercial antimicrobial drugs commonly used in the treatment of infectious diseases [10,11]. With the emergence of multidrug-resistant organisms, there are few or no treatment options for infections with certain microorganisms [12,13] as bacteria are resistant to 21 different antibiotics and each isolate is on average resistant to 7–8 antibiotics [14]. Under these circumstances where treatment becomes challenging, physicians have requested clinical laboratory to assess the adequacy of therapy [15–17], especially, when bactericidal antimicrobial agent therapy is considered necessary [17].

As a result, the need for new antimicrobial agents becomes greater than ever [18] and development of such agents faces significant challenges causing some pharmaceutical companies to curtail or abandon researches on anti-infective agents [19–21] while the cost of pharmaceutical research and development becomes high [22] and a number of factors make antimicrobial agents less economically attractive targets for development of other drug classes [23]. To this end, plants with related bioactive compounds varying in potency [24,25] and antimicrobial activity have attracted great attention [26–30], concurrent uses of pharmaceuticals with herbal remedies by laymen in self-medications are reported [31–33] and potential interactions of antibiotics with allopathic treatment and herbal remedies of various infections are inevitable. While the beneficial effects are expected as synergistic amplification or diminishment of possible adverse side effects, these combined bioactive compounds in plants prevented the gradual decline in efficacy frequently observed when single drugs are used over a long period of time [34]. Although South Africa’s rich plant biodiversity, with over 20,000 different species, is a great source of interest to the scientific community [35] and one way to prevent antibiotic resistance of pathogenic species is by using new compounds that are not based on existing synthetic antimicrobial agents [36] in a global village with ineffective synthetic antibiotics, investigating medicinal plants combined with synthetic or orthodox medicines against bacteria of clinical relevance might not be an invaluable effort. Medicinal plants, an indispensable part of the traditional medicine practiced all over the world because of low costs, easy access and ancestral experience [37], have many traditional claims which included the treatment of ailments of infectious origin [38]. They are an important source of new drugs, new drug leads and new chemical entities [39] to which pathogens are not resistant [40].

The pantropical genus *Acacia* (Fabaceae) includes over 1200 species [41,42] of which *Acacia mearnsii* De Wild is a member. *A. mearnsii* is a fast growing leguminous tree indigenous to Argentina but was introduced to South Africa over 150 years ago primarily for tanning industry [43]. The ethnobotanical studies showed it is a medicinal plant essential in the treatment of microbial infections in South Africa. While there is a dearth of information on its pharmacological importance, Olajuyigbe and Afolayan [44] indicated that the ethanolic and aqueous extract of *A. mearnsii* had a significant antibacterial activity. However, as a further study in cognizance of challenges of multidrug resistant bacteria, its antibacterial effect in combination with different first-line antibiotics commonly used against infectious agents needs to be investigated. Hence, to forestall drug-herbal interaction of clinical importance and indicate the degree of efficacy of these potential combinations to avert unexpected side effects, prolonged use of antibacterial agents and development of microbial resistance, this study was aimed at investigating the influence of combining methanolic extract of *A. mearnsii* with some of the commonly used antibiotics on the susceptibility of resistant bacteria of clinical importance.

## 2. Results and Discussion

From this study, the extract, antibiotics and their combinations exhibited significant antibacterial activities as shown in Table 1. With the exception of metronidazole (Met) not inhibiting any of the bacteria and erythromycin (Ery) that inhibited *E. faecalis* (ATCC 29212), *K. pneumoniae* (ATCC 10031) and *S. sonnei* (ATCC 29930) only, the bacterial inhibition zones produced by the methanolic extract ranged between 15 and 19 ± 1.0 mm, those of antibiotics ranged from 13 to 31 ± 1.0 mm while those of their combinations ranged between 13 and 29 ± 1.0 mm. While amoxicillin (Amx) inhibited all the bacteria to produce zones of inhibition ranging between 13 and 30 ± 1.0 mm, tetracycline (Tet) inhibited eight of the ten bacterial isolates, except *S. aureus* (ATCC 6538) and *P. vulgaris* KZN, with zones of inhibition ranging from 19 to 22 ± 1.0 mm. The zones of inhibition from chloramphenicol (Chl), nalidixic acid (Nal), and kanamycin (Kan) ranged between 17 and 25 ± 1.0 mm and ciprofloxacin (Cip) had zones of inhibition ranging between 19 and 31 ± 1.0 mm.

All the antibiotics and their combinations with the extract were more active than the extract. Apart from *S. aureus* (ATCC 6538), *P. vulgaris* (ATCC 6830) and bacteria not inhibited by metronidazole (Met) and erythromycin (Ery), the susceptibility of other bacteria indicated that zones of inhibition from antibiotics were wider in size than those of methanolic extract. The zones of inhibition produced by the antibacterial combinations, however, varied in size and were mostly wider than those obtained from either the extract or the antibiotics. In all the bacteria tested, extract combined with erythromycin (AE) had average zones of inhibition of 18 to 23 ± 1.0 mm. Extract combined with tetracycline (AT) had zones of inhibition ranging between 17 and 24 ± 1.0 mm. While that of extract combined with metronidazole (AM) ranged 15 to 21 ± 1.0 mm, those of extract combined with the amoxicillin (AA) were from 16 to 25 ± 1.0 mm. In the combined extract and erythromycin (AE), *B. subtilis* KZN, *E. faecalis* (ATCC 29212), *K. pneumoniae* (ATCC 10031) and *S. sonnei* (ATCC 29930) had the widest zones of inhibition equal to or greater than 20 ± 1.0 mm, others were less than 20 ± 1.0 mm. In extract combined with tetracycline (AT), *S. aureus* (ATCC 6538), *P. vulgaris* KZN and *E. cloacae* (ATCC 13047) had zones of inhibition less than 20 ± 1.0 mm, others had theirs being equal to or greater than 20 ± 1.0 mm. While *E. faecalis* KZN and *S. sonnei* (ATCC 29930) had zones of inhibition higher than 20 ± 1.0 mm in extract combined with metronidazole (AM), *E. coli* (ATCC 25922), *P. vulgaris* KZN, *E. faecalis* KZN, *E. cloacae* (ATCC 13047), *P. vulgaris* (ATCC 6830) and *S. sonnei* (ATCC 29930) had their zones of inhibition higher than 20 ± 1.0 mm in the combination of extract and amoxicillin (AA). AC exhibited highest antibacterial activity with the biggest zones of inhibition sizes, followed by AK, while other combinations exhibited varied degree of antibiotics activity. Based on the percentage of the number of bacteria with zones of inhibition equal to or greater than 20 ± 1.0 mm, the effect of combining the extract with the different antibiotics showed the following order: AK (100%) > ACh (90)% > AC = AT (70%) > AA (60%) > AN (50%)> AE (40%) > AM (20%).

In comparison to other combinations against a particular bacterium, AK was the most active against *S. aureus* (ATCC 6538), *P. vulgaris* KZN, *K. pneumoniae* (ATCC 10031), *P. vulgaris* (ATCC 6830) and *S. sonnei* (ATCC 29930). AC was the most active against *E. coli* (ATCC 25922), *B. subtilis* KZN, *E. cloacae* (ATCC 13047) and *E. faecalis* KZN. While the activity of AC was greater than those of AK, AE produced the highest inhibitory effect against *E. faecalis* (ATCC 29212) and AM produced the least inhibitory effect against the highest number of the test isolates. Of all the bacteria tested, *B. subtilis* KZN and *E. faecalis* KZN were the most inhibited bacteria by the AC. *E. faecalis* KZN was the most inhibited bacteria by the AN, AT and AM combinations. While *P. vulgaris* KZN was the most inhibited by AA, ACh and AK combinations, *E. faecalis* (ATCC 29212), and *K. pneumoniae* (ATCC 10031) and *S. sonnei* (ATCC29930) were the most inhibited bacteria by the AE combination.

Distinguishing synergistic from antagonistic interactions is of major importance for the development of improved strategies for the management of microbial infections. The *in vitro* antibacterial activity of the extract and the different antibiotics combinations was further assessed on the basis of the fractional inhibitory concentration (FIC) index representing the sum of the FICs (∑FICs) of each drug tested, where the FIC is determined for each drug by dividing the MIC of each drug when used in combination by the MIC of each drug when used alone. According to these MIC breakpoints of CLSI [45], the bacteria were classified as being susceptible, intermediate and resistant based on their susceptibility to each test antibiotic. The results showed that the MICs of the crude extract varied between 0.156 and 1.25 mg/mL and those of the antibiotics varied between 0.020 and 500 μg/mL (Table 2). When the evaluation criteria of Eliopoulos and Eliopoulos [46], Giertsen *et al.,* [47], Grytten *et al.* [48], Isenberg [49], Bhusal *et al.* [50], Petersen *et al.* [51] and Kamatou *et al.* [52] were applied to the results obtained and antibacterial combination resulting in ∑FIC ≤ 1.0 as synergism, 1.0 < ∑FIC ≤ 4 as indifference and ∑FIC > 4 as antagonism were considered, the antibacterial combinations showed synergistic interactions (61.25%), additivity/indifference (23.75%) and antagonism (15.0%) against the test organism. Considering synergism as ∑FIC ≤ 0.5, additivity/indifference as 5 < ∑FIC ≤ 4 and antagonism as ∑FIC > 4, the extract-antibiotics combinations indicated synergism (20%), additivity/indifference (71.25%) and antagonism (8.75%) (Table 3). The number of synergistic interaction between the AmM and the antibiotics against different bacteria in each treatment is in the following order: ACh > AA > AM ≥ AE > AT > AC = AN > AK. That is, ACh was synergistic against more bacteria, followed by AA, while AK was synergistic against the least number of the bacterial isolates. While AK had additive/indifferent effect against eight of the ten organisms, followed by AN and AT, others showed varied degree of additive/indifferent effects against different numbers of test bacteria. On the other hand, synergistic interaction was most expressed against *E. coli* (ATCC 25922) by AT, AM, AA, AC, ACh and AN, *S. aureus* (ATCC 6538) by AE, AM, AA, ACh and AK, *B. subtilis* KZN by AE, AT, AA, AN and ACh, *E. faecalis* KZN by AE, AM and AA, *K. pneumoniae* (ATCC 10031) by AE, AT, AN and ACh, *P. vulgaris* (ATCC 6830) by AE, AT, AM and ACh, *S. sonnei* (ATCC 29930) by AE, AT, AA and ACh, *E. faecalis* (ATCC 29212) by AM, AA and ACh, *Proteus vulgaris* KZN by AC and ACh. The differences in the resultant synergistic, indifferent and antagonistic interactions were due to the elevated MIC values obtained from the resistance of these bacteria to some of the antibiotics.

Due to the emergence of multidrug-resistant pathogens, treatment with antibacterial combinations, using two or more antibacterials has become commonplace [53]. In search of more effective chemotherapeutic agents for treating microbial infections, combination therapy becomes an important strategy as synergistic interactions can potentially increase efficacy, reduce toxicity, cure faster, prevent the emergence of resistance, and provide broader-spectrum of activity than monotherapy regimens [54]. It has been shown to be beneficial for several difficult-to-treat infections such as human immunodeficiency virus and mycobacterial infections, which do not respond well to single-drug therapy due to either lack of efficacy or rapid emergence of resistance [55]. Since drug-drug combinations are convenient models of additivity, their experimental properties can, then, give valuable insights into the significance of synergistic and antagonistic interactions of dissimilar drugs [56,57]. Hence, it is important to interact the first line antibiotics, used in this study, with medicinal plant materials in order to identify synergistic and/or antagonistic effects able to provide guidance for empirical use, reduce cost and duration of antimicrobial therapy.

Consequently, to identify synergistic or antagonistic antibacterial combinations capable of providing empirical use and treatment of poly-microbial infections where combination therapy is required [53], *in vitro* combination of plant materials with different antibiotics had been investigated against potential pathogens. As a result, several studies [58–62] showed that there are varied interactions between plant extracts and antibiotics. In agreement with these studies, our study demonstrated synergism, additivity/indifference and antagonism between methanolic extract of *Acacia mearnsii* and different classes of first line antibiotics. The synergistic effects indicated that the antibacterial combinations were more effective than the activity of the individual agents. The increase in the sizes of the zones of inhibition and the decreased MICs resulting from the extract and antibiotic combinations indicated the improved bactericidal potentials of the extract and the antibiotics as combined antibacterial agents. These variations were adjudged by Pei *et al.* [63] to have resulted from differences in mechanisms of action and variation of combined antibacterial action in the tested bacteria.

Although the extract combined with antibiotics, having different target sites, exhibit varied degree of antibacterial activity, the ability of these antibacterial combinations to inhibit resistant bacteria showed that they have either attacked different target sites singly or combined to overcome inherent resistant mechanisms in the isolates. One of the phytoconstituents, such as flavonoids or the polyphenols, may have interacted with the antibiotics to enhance its mechanisms of action at the target sites for which the antibiotic was designed. Since Cushnie *et al.* [64] indicated synergy between flavonoids and chemotherapeutics and Sato *et al.* [65] and Cushnie *et al.* [64] reported antimicrobial and resistance modulating potentials of flavonoids and polyphenols, combining the methanolic extract with the antibiotics could have altered the inherent resistant properties in the bacteria to be more effective [65–68]. In addition, the initial role of each phytoconstituent in antimicrobial activity and in their combination with antibiotics may not be underestimated. Aromatic planar quaternary alkaloids in extracts interchelate with DNA [69]. Lipophilic flavonoids disrupt microbial membranes [70]. Tannins precipitate microbial protein [71]. Saponins having detergent properties serve as lytic agents [72]. These phytochemicals in the extract could have acted singly or synergistically with other compounds for effective antibacterial activities. While the double attack of extracts with antibiotics on different target sites was noted [73], the phytoconstituents coupled with the antibiotics targeting specific sites could have formed a complex with enhanced antibacterial properties. On the other hand, the cell membrane, vital for any microorganism, is the primary target for most antibacterial agents [74]. Against resistant bacteria, a component of the phytoconstituents could have acted singly to cause membrane disruption or translocate through the membrane to a target receptor inside the cell to pave ways for each of these antibiotics to reach their target sites of action to upshot their concerted activities [75] while blocking the inhibitory effects of protective enzymes [76–78]. While Zhao *et al.* [79] reported that some phytochemicals can improve the *in vitro* activity of some peptidoglycan inhibiting antibiotics by attacking the same site in the cell wall, Esinome *et al.* [73] showed that polyphenols couple with β-lactams could enhance antibacterial activity to disrupt transpeptidation of the cell membrane. While these may account for the varied degree of antibacterial activities of the different combinations, it could account for the differences in the rate at which the antibiotics get to their target sites.

Though efflux pumps have been associated with resistance mechanisms in bacteria, resistance modifying agents [80] and the ability of combinations between extracts and antibiotics [78,81] to inhibit these resistance mechanisms [82] have been reported. In agreement with these earlier reports, the ability of methanolic extract combined with each antibiotic to inhibit bacteria that exhibited low susceptibility and high MICs showed that these combinations could mediate in bacterial resistance and serve as a source of plant derived natural products with antibiotic resistance-modifying activity to be used against multi-drug resistant bacteria in hospital and community acquired infections.

## 3. Experimental Section

### 3.1. Collection of Plant Material

The bark materials of *Acacia mearnsii* De Wild were collected in August 2010, from the plant growing within the University of Fort Hare campus in Alice, South Africa. The plant was authenticated in the Department of Botany and a voucher specimen (OLAJ Med 2010/01) was prepared and deposited in the Griffen Herbarium of the University.

### 3.2. Extract Preparation

The bark sample was air-dried at room temperature and pulverized using a milling machine. The extract of the bark material was prepared in accordance to the description of Basri and Fan [83]. About 100 g of the pulverized sample was extracted with 500 mL of methanol for 48 h with shaking (Stuart Scientific Orbital Shaker, UK). The extract was filtered through Whatman No. 1 filter paper and concentrated under reduced pressure at 40 °C using a rotary evaporator (Laborota 4000 efficient, Heldolph, Germany). The crude extract collected was allowed to dry at room temperature to a constant weight of 18.7 g. The extract was redissolved in dimethylsulfoxide (DMSO) to the required concentrations for bioassay analysis.

The reconstituted extract solution was sterilized by filtering through 0.45 μm membrane filter and tested for sterility after membrane filtration by introducing 2 mL of the extract into 10 mL of sterile nutrient broth before being incubated at 37 °C for 24 h. A sterile extract was indicated by the absence of turbidity in the broth after the incubation period.

### 3.3. Bacterial Strain

The bacteria used in this study included *Staphylococcus aureus* (ATCC 6538), *Enterococcus faecalis* (ATCC 29212), *Escherichia coli* (ATCC 25922), *Baciilus subtilis* KZN, *Proteus vulgaris* KZN, *Enterococcus faecalis* KZN, *Enterobacter cloacae* (ATCC 13047), *Klebsiella pneumoniae* (ATCC 10031), *Proteus vulgaris* (ATCC 6830) and *Shigella sonnei* (ATCC 29930). These organisms were obtained from the Department of Biochemistry and Microbiology, University of Fort Hare, Alice, South Africa. The antibacterial assays were carried out using Mueller-Hinton II Agar (Biolab) and broth. The inocula of the test bacteria were prepared using the colony suspension method [84]. Colonies picked from 24 h old cultures grown on nutrient agar were used to make suspensions of the test organisms in saline solution to give an optical density of approximately 0.1 at 600 nm. The suspension was then diluted 1:100 by transferring 0.1 mL of the bacterial suspension to 9.9 mL of sterile nutrient broth before being used.

### 3.4. Antibiotics Used in This Study

Antibiotic powders of amoxicillin, chloramphenicol, ciprofloxacin, erythromycin, tetracycline hydrochloride, metronidazole, kanamycin and nalidixic acid were used. Stock antibiotic solutions were prepared and dilutions made according to the CLSI (Clinical Laboratory Standardization Institute) method or manufacturer’s recommendations [85,86].

### 3.5. Antibiotic Susceptibility Testing—Agar Diffusion Method

Each of the isolates was standardized using colony suspension method. Each strain’s suspension was matched with 0.5 McFarland standards to give a resultant concentration of 1.5 × 10^8^ cfu/mL. The antibiotic susceptibility testing was determined using the modified Kirby–Bauer diffusion technique [87] by swabbing the Mueller-Hinton agar (MHA) (Oxoids UK) plates with the resultant saline suspension of each strain. Wells were then bored into the agar medium with heat sterilized 6 mm cork borer. The wells were filled with 100 μL of different concentrations prepared for the methanolic extract alone, antibiotics alone and their combinations taking care not to allow spillage of the solutions onto the surface of the agar. The plates were allowed to stand for at least 30 min before being incubated at 37 °C for 24 h [88]. The determinations were done in duplicate. After 24 h of incubation, the plates were examined for zones of inhibition [89]. The diameter of the zones of inhibition produced by the extract alone, antibiotic alone and their combinations were measured and interpreted using the CLSI zone diameter interpretative standards [45].

### 3.6. Determination of Minimal Inhibitory Concentration (MIC)

The minimum inhibitory concentrations (MICs) for the extract and the antibiotics under study were determined in duplicate by the macrobroth dilution method in Mueller-Hinton broth according to CLSI (Clinical Laboratory Standardization Institute) [86]. To determine the MICs of each antibiotic, the concentrations used for each of the antibiotics (0.0019–500) μg/mL and those of extract (0.078125–5) mg/mL were prepared by serial dilution in Mueller-Hinton broth. To determine their combinatorial effects, combinations of different concentrations ranging from ½ × MIC to 8× MIC of each of the antibiotics and the extract were used. The tubes were inoculated with 100 μL of each of the bacterial strains. Blank Mueller-Hinton broth was used as negative control. The bacterial containing tubes were incubated aerobically at 37 °C for 24 h. Each combination assay was performed two times. The MIC was defined as the lowest dilution that showed no growth in the Mueller-Hinton broth.

### 3.7. Checkerboard Assay

The interactions between the extract and the antibiotics were determined using the checkerboard as previously described [51]. The range of drug concentration used in the checkerboard assay was such that the dilution range encompassed the MIC for each drug used in the analysis. The fractional inhibitory concentration (FIC) was derived from the lowest concentrations of the extract and the antibiotics in combination permitting no visible growth of the test organisms in the Mueller-Hinton broth after incubation for 24 h at 37 °C [90]. FIC indices were calculated using the formula: FIC index = (MIC of extract in combination/MIC of extract alone) + (MIC of antibiotics in combination/MIC of antibiotics alone). In antimicrobial combination, Eliopoulos and Eliopoulos [46], Isenberg [47] and Petersen *et al.* [51] defined synergy as ∑FIC ≤ 0.5, additivity as 5 < ∑FIC ≤ 1, indifference as 1 < ∑FIC ≤ 4 and antagonism as ∑FIC > 4. Giertsen *et al.* [47], Grytten *et al*. [48], and Kamatou *et al.* [52] defined synergy to occur when ∑FIC < 1.0, additivity when ∑FIC = 1.0 and antagonism when ∑FIC > 1.0. Bhusal *et al.* [50] also showed synergism as ∑FIC ≤ 0.75, indifference as 0.75 < ∑FIC ≤ 4, and antagonism as ∑FIC > 4. This implies that synergy by the checkerboard method may be defined as FIC ≤ 0.5 or FIC ≤ 1.

## 4. Conclusions

The combinations of antibacterial agents demonstrating *in vitro* synergism against infectious agents are most likely to be a means of achieving pragmatic and effective treatment for bacterial infection especially in patients with infections difficult to treat. Since the development of new classes of antibacterial agents is of paramount importance, the crude methanolic extract of *A. mearnsii* showed potential synergy on being combined with some antibiotics against resistant bacteria of clinical importance and suggested that varied degree of effective therapy will be more attained with these antibacterial combinations. Hence, assessing the therapeutic potentials of this plant allows us to know how best it can be used in the treatment of diseases, especially, when the synergistic competency between plants and standard antibiotics is highly required to complement effective therapy. This work has indicated the potential of this plant as a source of resistance modulating and novel chemotherapeutic agents for the production of synthetically improved therapeutic agents that could be combined with antibiotics to enhance their antimicrobial activities. Further works including the isolation of the bioactive phytochemical compounds, investigation of the mechanisms of actions and *in vivo* studies to validate the therapeutic potentials of these combinations are ongoing in our Research Centre.

## Figures and Tables

**Table 1:** Average zones of inhibition (±1.0 mm) from AMM alone, antibiotics alone and their combinations concentrations.

	Average Zones of Inhibition (±1.0 mm) Produced by Methanolic Extract (AmM) Alone, Antibiotics Alone and Their Combinations																	
Bacteria Used	A	B	C	D	E	F	G	H	I	J	K	L	M	N	P	Q	R	S
*S. aureus* (ATCC 6538)	17 ± 0.58	0	18 ± 0.58	0	17 ± 0.58	0	15 ± 0.58	13 ± 0.58	18 ± 0.58	17 ± 0.00	22 ± 1.00	23 ± 0.58	17 ± 0.58	19 ± 0.58	21 ± 1.00	18 ± 0.00	25 ± 1.00	24 ± 0.58
*E. faecalis* (ATCC 29212)	15 ± 0.58	22 ± 0.58	23 ± 0.00	19 ± 0.00	20 ± 0.58	0	16 ± 0.58	20 ± 0.58	18 ± 1.00	15 ± 0.58	20 ± 1.00	19 ± 0.58	17 ± 1.00	18 ± 0.58	23 ± 1.00	20 ± 1.00	21 ± 0.58	22 ± 0.58
*E. coli* (ATCC 25922)	16 ± 0.00	0	18 ± 0.58	20 ± 0.58	22 ± 0.58	0	15 ± 0.58	22 ± 1.00	20 ± 0.58	19 ± 0.58	30 ± 1.00	27 ± 0.58	25 ± 0.00	29 ± 0.58	22 ± 0.00	21 ± 0.58	20 ± 1.00	21 ± 0.58
*B. subtilis* KZN	18 ± 0.00	0	20 ± 0.58	19 ± 0.58	21 ± 0.58	0	19 ± 0.58	24 ± 1.53	16 ± 0.00	18 ± 0.58	26 ± 1.00	29 ± 0.58	20 ± 0.58	22 ± 0.58	17 ± 0.58	20 ± 0.58	22 ± 0.58	21 ± 0.58
*P. vulgaris* KZN	16 ± 1.00	0	18 ± 0.58	0	19 ± 0.00	0	15 ± 1.00	30 ± 1.53	25 ± 1.00	17 ± 0.58	27 ± 0.58	25 ± 0.00	21 ± 0.58	19 ± 0.00	23 ± 0.58	24 ± 0.58	25 ± 0.58	26 ± 0.00
*E. faecalis* KZN	18 ± 0.58	0	18 ± 0.00	21 ± 0.58	24 ± 0.58	0	20 ± 0.58	23 ± 0.58	22 ± 0.00	18 ± 0.00	30 ± 1.00	29 ± 0.58	24 ± 0.58	25 ± 0.58	23 ± 0.58	21 ± 0.58	22 ± 0.58	25 ± 0.58
*E. cloacae* (ATCC 13047)	17 ± 0.00	0	17 ± 1.15	17 ± 1.52	19 ± 0.58	0	13 ± 0.58	19 ± 1.15	22 ± 1.00	16 ± 1.00	27 ± 1.53	24 ± 0.58	22 ± 0.58	20 ± 0.58	22 ± 0.58	20 ± 0.58	22 ± 0.58	21 ± 1.00
*K. pneumoniae* (ATCC 10031)	17 ± 0.58	23 ± 0.58	23 ± 1.53	20 ± 1.00	22 ± 0.58	0	17 ± 0.58	18 ± 0.58	18 ± 1.15	17 ± 0.00	19 ± 1.53	22 ± 0.58	18 ± 0.00	19 ± 0.58	21 ± 0.58	22 ± 0.58	21 ± 0.58	24 ± 1.00
*P. vulgaris* (ATCC 6830)	18 ± 0.00	0	19 ± 0.00	21 ± 1.53	23 ± 1.15	0	15 ± 0.58	17 ± 0.58	21 ± 0.58	18 ± 0.00	22 ± 1.00	17 ± 0.58	19 ± 0.58	19 ± 0.58	23 ± 0.58	20 ± 0.58	21 ± 0.00	21 ± 1.15
*S. sonnei* (ATCC 29930)	19 ± 0.58	22 ± 1.00	23 ± 0.58	22 ± 1.53	23 ± 0.58	0	21 ± 0.58	21 ± 0.58	22 ± 0.58	19 ± 0.58	20 ± 0.58	18 ± 0.00	19 ± 0.58	21 ± 0.58	24 ± 1.00	21 ± 0.58	22 ± 0.00	23 ± 0.00

A = Methanol extract (AmM) (10 mg/mL); B = Erythromycin (Ery) (25 μg/mL); C = AmM (10 mg/mL) + Ery (25 μg/mL); D = Tetracycline (Tet) (62.5 μg/mL); E = AmM (10 mg/mL) + Tet (62.5 μg/mL); F = Metronidazole (Met) (125 μg/mL); G = AmM (10 mg/mL) + Met (125 μg/mL); H = Amoxicillin (Amx) (62.5 μg/mL); I = AmM (10 mg/mL) + Amx (62.5 μg/mL); J = Methanol extract (AmM) (10 mg/mL); K = Ciprofloxacin (Cip) (2.5 μg/mL); L = AmM (10 mg/mL) + Cip (2.5 μg/mL); M = Nalidixic acid (Nal) (125 μg/mL); N = AmM (10 mg/mL) + Nal (125 μg/mL); P = Chloramphenicol (Chl) (25 μg/mL); Q = AmM (10 mg/mL) + Chl (25 μg/mL); R = Kanamycin (Kan) (125 μg/mL); S = AmM (10 mg/mL) + Kan (125 μg/mL).

**Table 2:** The minimum inhibitory concentrations (MICs) of the extract and the antibiotics used.

	Minimum Inhibitory Concentrations								

	AmM (mg/mL)	Ery	Tet	Met	Amx	Cip	Nal	Chl	Kan

		(μg/mL)							
*S. aureus* (ATCC 6538)	0.313	6.250 (R)	7.813 (R)	15.625 (R)	31.250 (R)	0.020 (S)	31.250 (R)	1.953 (S)	1.953 (S)
*E. faecalis* (ATCC 29212)	1.250	0.195 (S)	3.906 (S)	31.250 (R)	3.906 (S)	0.313 (S)	31.250 (R)	1.953 (S)	125.000 (R)
*E. coli* (ATCC 25922)	0.313	1.563 (R)	1.953 (S)	31.250 (R)	7.813 (I)	0.039 (S)	1.953 (S)	3.906 (S)	125.000 (R)
*B. subtilis* KZN	1.250	6.250 (R)	0.977 (S)	31.250 (R)	62.500 (R)	0.020 (S)	7.813 (S)	3.906 (S)	3.906 (S)
*P. vulgaris* KZN	0.313	12.500 (R)	7.813 (R)	62.500 (R)	0.488 (S)	0.313 (S)	62.500 (R)	0.977 (S)	7.8125 (S)
*E. faecalis* KZN	0.156	12.500 (R)	31.250 (R)	15.625 (R)	0.976 (S)	0.313 (S)	62.500 (R)	31.250 (R)	250.000 (R)
*E. cloacae* (ATCC 13047)	0.625	6.250 (R)	15.625 (R)	31.250 (R)	500.000 (R)	0.156 (S)	62.500 (R)	1.953 (S)	62.500 (R)
*K. pneumoniae* (ATCC 10031)	0.313	0.195 (S)	0.488 (S)	31.250 (R)	0.977 (S)	0.039 (S)	3.906 (S)	1.953 (S)	15.625 (S)
*P. vulgaris* (ATCC 6830)	0.313	25.000 (R)	15.625 (R)	62.250 (R)	250.000 (R)	0.156 (S)	1.953 (S)	7.813 (I)	31.250 (R)
*S. sonnei* (ATCC 29930)	0.156	0.391 (S)	1.953 (S)	62.250 (R)	500.000 (R)	0.020 (S)	15.563 (I)	1.953 (I)	31.250 (R)

**Table 3:** Fractional inhibitory concentrations of different combination of the extract and the antibiotics.

	Fractional Inhibitory Concentration			Remarks	Fractional Inhibitory Concentration			Remarks
	
	FICI (AmM)	FICI (Ery)	FICI index		FICI (AmM)	FICI (Tet)	FICI index	
*S. aureus* (ATCC 6538)	0.5	0.25	0.75	Synergy	0.5	0.5	1	Indifference
*E. faecalis*(ATCC 29212)	0.0156	1	1.0156	Indifference	0.125	1	1.125	Indifference
*E. coli* (ATCC 25922)	0.25	0.5	0.75	Synergy	0.0624	0.25	0.3125	Synergy
*B. subtilis* KZN	0.25	0.5	0.75	Synergy	0.0156	0.5	0.5156	Synergy
*P. vulgaris* KZN	1	0.25	1.25	Indifference	0.5	0.5	1	Indifference
*E. faecalis* KZN	0.5	0.0625	0.5625	Synergy	2	0.5	2.5	Indifference
*E. cloacae* (ATCC 13047)	0.5	0.5	1	Indifference	0.5	0.5	1	Indifference
*K. pneumoniae* (ATCC 10031)	0.0312	0.5	0.5312	Synergy	0.0156	0.25	0.2656	Synergy
*P. vulgaris* (ATCC 6830)	0.5	0.0625	0.5625	Synergy	0.5	0.25	0.75	Synergy
*S. sonnei* (ATCC 29930)	0.015625	0.0625	0.0781	Synergy	0.0312	0.0625	0.0937	Synergy

	**Fractional Inhibitory Concentration**			**Remarks**	**Fractional Inhibitory Concentration**			**Remarks**
	
	**FICI (AmM)**	**FICI (Met)**	**FICI index**		**FICI (AmM)**	**FICI (Amx)**	**FICI index**	

*S. aureus* (ATCC 6538)	0.5	0.25	0.75	Synergy	0.5	0.125	0.625	Synergy
*E. faecalis* (ATCC 29212)	0.125	0.125	0.25	Synergy	0.0078	0.0625	0.0703	Synergy
*E. coli* (ATCC 25922)	0.25	0.0625	0.3125	Synergy	0.25	0.25	0.5	Synergy
*B. subtilis* KZN	0.5	0. 5	1.0	Indifference	0.5	0.25	0.75	Synergy
*P. vulgaris* KZN	1	0.25	1.25	Indifference	0.125	2	2.125	Indifference
*E. faecalis* KZN	0.5	0.125	0.625	Synergy	0.0625	0.25	0.3125	Synergy
*E. cloacae* (ATCC 13047)	0.5	0.25	0.75	Synergy	0.5	0.015625	0.5156	Synergy
*K. pneumoniae* (ATCC 10031)	1	0.25	1.25	Indifference	0.25	2	2.25	Indifference
*P. vulgaris* (ATCC 6830)	0.5	0.0625	0.5625	Synergy	1	0.03125	1.03125	Indifference
*S. sonnei* (ATCC 29930)	2	0.25	2.25	Indifference	0.015625	0.000122	0.0157	Synergy

	**Fractional Inhibitory Concentration**			**Remarks**	**Fractional Inhibitory Concentration**			**Remarks**
	
	**FICI (AmM)**	**FICI (Cip)**	**FICI index**		**FICI (AmM)**	**FICI (Nal)**	**FICI index**	

*S. aureus* (ATCC 6538)	0.25	4	4.25	Antagonistic	1	0.25	1.25	Indifference
*E. faecalis* (ATCC 29212)	0.25	1	1.25	Indifference	0.5	0.5	1.0	Indifference
*E. coli* (ATCC 25922)	0.0625	0.5	0.5625	Synergy	0.125	0.5	0.625	Synergy
*B. subtilis* KZN	0.0625	4	4.0625	Antagonistic	0.3125	0.125	0.4375	Synergy
*P. vulgaris* KZN	0.25	0.25	0.5	Synergy	2	0.25	2.25	Indifference
*E. faecalis* KZN	1	0.5	1.5	Indifference	4	0.25	4.25	Antagonistic
*E. cloacae* (ATCC 13047)	0.125	0.5	0.625	Synergy	1	0.25	1.25	Indifference
*K. pneumoniae* (ATCC 10031)	0.25	2	2.25	Indifference	0.25	0.5	0.75	Synergy
*P. vulgaris* (ATCC 6830)	0.5	1	1.5	Indifference	1	4	5	Antagonistic
*S. sonnei* (ATCC 29930)	2	16	18	Antagonistic	2	0.5	2.5	Indifference

	**Fractional Inhibitory Concentration**			**Remarks**	**Fractional Inhibitory Concentration**			**Remarks**
	
	**FICI (AmM)**	**FICI (Chl)**	**FICI index**		**FICI (AmM)**	**FICI (Kan)**	**FICI index**	

*S. aureus* (ATCC 6538)	0.125	0.5	0.625	Synergy	0.125	0.5	0.625	Synergy
*E. faecalis* (ATCC 29212)	0.03125	0.5	0.53125	Synergy	1	0.25	1.25	Indifference
*E. coli* (ATCC 25922)	0.125	0.25	0.375	Synergy	1	0.0625	1.0625	Indifference
*B. subtilis* KZN	0.015625	0.125	0.140625	Synergy	0.125	1	1.125	Indifference
*P. vulgaris* KZN	0.0625	0.5	0.5625	Synergy	0.5	0.5	1.0	Indifference
*E. faecalis* KZN	2	0.25	2.25	Indifference	8	0.125	8.125	Antagonistic
*E. cloacae* (ATCC 13047)	0.5	4	4.5	Antagonistic	2	0.5	2.5	Indifference
*K. pneumoniae* (ATCC 10031)	0.125	0.5	0.625	Synergy	1	0.5	1.5	Indifference
*P. vulgaris* (ATCC 6830)	0.25	0.25	0.5	Synergy	1	0.25	1.25	Indifference
*S. sonnei* (ATCC 29930)	0.125	0.25	0.375	Synergy	2	0.25	2.25	Indifference
